# Machine Learning Techniques Associated With Infrared Thermography to Optimize the Diagnosis of Bovine Subclinical Mastitis

**DOI:** 10.1155/vmi/5585458

**Published:** 2025-02-08

**Authors:** Raul Costa Mascarenhas Santana, Edilson da Silva Guimarães, Fernando David Caracuschanski, Larissa Cristina Brassolatti, Maria Laura da Silva, Alexandre Rossetto Garcia, José Ricardo Macedo Pezzopane, Teresa Cristina Alves, Patrícia Tholon, Marcos Veiga dos Santos, Luiz Francisco Zafalon

**Affiliations:** ^1^School of Agricultural and Veterinary Sciences, São Paulo State University (UNESP), Jaboticabal, São Paulo, Brazil; ^2^Embrapa Southeastern Livestock, São Carlos, São Paulo, Brazil; ^3^Central Paulista University Center (UNICEP), São Carlos, São Paulo, Brazil; ^4^School of Veterinary Medicine and Animal Science, University of São Paulo (FMVZ-USP), São Paulo, São Paulo, Brazil

**Keywords:** dairy cattle, Extreme Gradient Boosting, robotic milking system

## Abstract

Bovine subclinical mastitis (SCM) is the costliest disease for the dairy industry. Technologies aimed at the early diagnosis of this condition, such as infrared thermography (IRT), can be used to generate large amounts of data that provide valuable information when analyzed using learning techniques. The objective of this study was to evaluate and optimize the use of machine learning by applying the Extreme Gradient Boosting (XGBoost) algorithm in the diagnosis of bovine SCM, based on udder thermogram analysis. Over 14 months, a total of 1035 milk samples were collected from 97 dairy cows subjected to an automatic milking system. Somatic cell counts were performed by flow cytometry, and the health status of the mammary gland was determined based on a cutoff of 200,000 cells/mL of milk. The attributes analyzed collectively included air temperature, relative humidity, temperature-humidity index, breed, body temperature, teat dirtiness score, parity, days in milk, mammary gland position, milk yield, electrical conductivity, milk fat, coldest and hottest points in the mammary gland region of interest, average mammary gland temperature, thermal amplitude, and the difference between the average temperature of the region of interest and the animal's body temperature, as well as the microbiological evaluation of the milk. Using the XGBoost algorithm, the most relevant variables for solving the classification problem were identified and selected to construct the final model with the best fit and performance. The best area under the receiver operating characteristic curve (AUC: 0.843) and specificity (Sp: 93.3%) were obtained when using all thermographic variables. The coldest point in the region of interest was considered the most important for decision making in mastitis diagnosis. The use of XGBoost can enhance the diagnostic capability for SCM when IRT is employed. The developed optimized model can be used as a confirmatory mechanism for SCM.

## 1. Introduction

Mastitis is the costliest disease for the dairy industry due to losses caused by decreased milk production and quality, increased veterinary costs, reduced productive life, and higher expenses for animal replacement. The subclinical form of the disease not only reduces production but also impacts the nutritional quality of milk [1, 2]. The subclinical mastitis (SCM) can be up to 40 times more common than clinical mastitis, is more difficult to identify, and has a greater economic impact [3].

Due to deficiencies in the application of preventive measures and the limited use of diagnostic techniques, most subclinical diseases remain unidentified until clinical symptoms appear. This prolongs the negative effects on the health and performance of dairy cows [4]. Therefore, methods aiming for the rapid detection of SCM with high diagnostic efficiency are essential for disease control. Early identification can help prevent the deterioration of udder health and improve the efficiency of dairy operations [5].

The infrared thermography (IRT) is a technology with the potential to be used as a screening method for detecting animals with localized inflammation. Objects with temperatures above absolute zero emit infrared radiation in the form of waves proportional to their body temperature. The thermal camera can capture the radiation emitted by the target animals and the environment, generating a matrix of thermal data represented by thermograms, which can be interpreted to determine the thermal condition of an animal or part of its body [6].

In addition to being a fast, effective, and noninvasive tool, IRT has been described as a sensitive technique for detecting small temperature variations on the surface of the udder caused by SCM [1, 7]. However, its use in applications aimed at diagnosing SCM has the potential for improvement, although it still requires refinement and development [8]. It is assumed that the diagnostic accuracy of IRT can be enhanced when used in combination with other diagnostic methods, taking into account factors that limit the use of thermal cameras [1], such as direct sunlight, humidity, and the dirtiness of the evaluated organ, as well as specific meteorological conditions [9].

When used in real time in the daily control of milking systems, IRT can generate a large number of thermograms for analysis and compose large databases. Analyzing this information can aid the producer in decision making. Developing techniques for processing thermal data would facilitate diagnostic interpretation through more precise information [6], as well as advance control procedures and management measures.

Currently, some health disorders in dairy cattle can be identified and predicted through the use of machine learning algorithms, which integrate and analyze data from various sources [4]. Machine learning techniques can be used to translate large volumes of data (“Big Data”) into valuable information [10]. In this context, machine learning techniques can be applied to develop statistical models for selecting the main predictive characteristics of SCM. The process of extracting information from datasets, previously unknown and potentially useful, aims to identify regularities and patterns, surpassing common multivariate statistical methods in large-scale studies [3].

Supervised learning techniques include neural networks, decision trees, Naive Bayes models, or support vector machines, in which the data used to develop the model are labeled [10]. However, successful applications of machine learning in various fields result from two important factors: the use of effective statistical models that capture the complex interdependencies of the data, and scalable learning systems that learn the model of interest from large datasets [11].

The Extreme Gradient Boosting (XGBoost) algorithm is a scalable decision tree boosting system widely used in data science that provides state-of-the-art results for a variety of problems [11]. It has built-in parameters capable of addressing important optimization issues such as regularization and overfitting-underfitting. Its operation involves evaluating effectiveness in classification problems by estimating accuracy and determining the area under the receiver operating characteristic curve (AUC) for a set of hyperparameter values that require adjustments [12].

The analysis of databases from digital technologies such as IRT using machine learning techniques is disruptive and promising. It has the potential to generate information for decision making, aiming at technical intervention and reducing the negative effects of SCM in dairy cows. Therefore, the objective of this study was to evaluate and improve the use of machine learning by applying the XGBoost algorithm in the diagnosis of bovine SCM through the development of image analysis strategies for mammary glands generated by IRT.

## 2. Materials and Methods

### 2.1. Experiment Location, Period, and Animals

The experiment was conducted at Embrapa Southeastern Livestock in São Carlos, Brazil (22°01′S 47°53′W, altitude 856 m above sea level), from October 2021 to December 2022. Ninety-seven dairy cows, Holstein and Holstein vs. Jersey crossbreeds, with varying degrees of lineage, between 15 and 275 days in lactation, were voluntarily milked using the DeLaval VMS V300 automatic milking system. The animals were raised on a *Megathyrsus* (syn. *Panicum*) *maximum* Jacques cv. BRS Tamani pasture, maintained under irrigation and overseeded with oats (*Avena byzantina* Koch, cv. IPR Esmeralda) in the fall, and on a *Urochloa* (syn. *Brachiaria*) *brizantha* (Hochst ex A. Rich.) Stapf cv. BRS Paiaguas pasture, interspersed with *Corymbia citriodora* trees. They received concentrate automatically during milking according to milk production. During the winter, they were supplemented with corn silage in troughs. Mineral mix and water were provided *ad libitum*.

### 2.2. Milking Environment

The environmental conditions of air temperature (AT) and relative humidity (RH) were continuously monitored. Data were collected using a Hobo U12-013 sensor installed in the milking cabin, positioned 20 cm above the cows' backs. The sensor was programmed to record readings every 10 s and provide average outputs every 5 min [13, 14]. The AT and RH were associated through modeling by applying the temperature-humidity index (THI), as described by Thom [15]. The environmental indicators were used to evaluate their influence on the diagnosis of SCM using IRT, providing an accurate understanding of the effect of the robotic milking microclimate.

### 2.3. Acquisition of Thermograms and Data Extraction

Thermographic images were acquired immediately before collecting milk samples for microbiological testing from the lactating cows and prior to milking. A thermal camera with a 640 × 480-pixel detector, equipped with a 25-degree fixed lens and an 11° × 9° telephoto lens, thermal sensitivity < 40 mK (< 0.04°C at 30°C AT), and a temperature range from −30°C to 350°C was used, adopting the manual focus function. Images of the mammary quarters were taken from a lateral approach, always from the right antimer. The emissivity was set to 0.98 [16].

For each mammary gland sample unit, at least three thermal images were acquired during the evaluation of each animal. As the images were generated while the animal was not restrained and was in the milking parlor of the voluntary system, not all images had the minimum attributes required for subsequent analysis (e.g., perfect orthogonality between the thermal sensor and the mammary gland). For this reason, the 4756 images included in the image database were subjected to a preliminary assessment of whether they met the requirements (orthogonality, focus adjustment, absence of intrusions or moisture on the mammary gland, etc.). A single thermogram was then selected for each analyzed mammary gland, again based on the largest field of view that allowed the region of interest (ROI) to be perfectly defined. This resulted in a total of 1031 thermograms, of which 40 records with partially missing data were removed during the data cleaning process to reduce bias and increase the accuracy of the analysis, resulting in 991 thermograms for machine learning.

**Figure 1:**
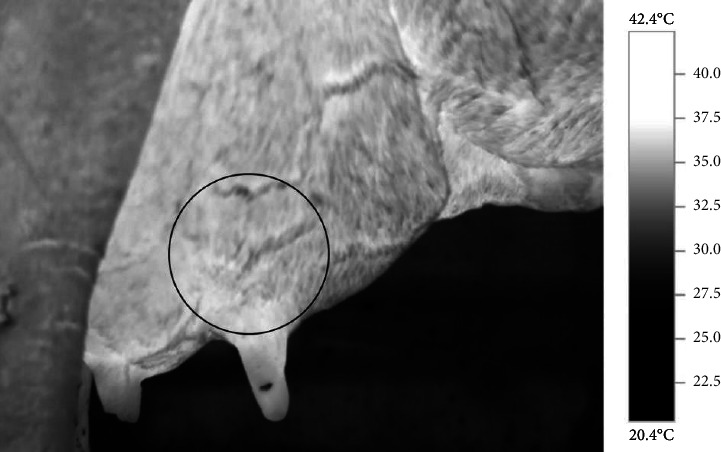
Thermographic image of the mammary gland of a lactating cow generated with the IRSoft software, evidencing the region of interest (circular delimitation). Image parametrized to a thermal scale of 20.4°C–42.4°C and using the greyscale color palette.

The thermograms were analyzed using the software IRSoft, Version 4.8 SP1 (Testo AG; Lenzkirch, Germany), available at https://www.testo.com/pt-BR/produtos/termografia-irsoft. To this end, a circular-shaped ROI was delimited in the mammary quarter, immediately above the teat of the gland under analysis (Figure 1), as recommended by Hovinen [17]. The variables of interest included the temperatures of the coldest point (cold spot), of the hottest point (hot spot), and the average temperature of the ROI (°C). The average surface temperature of the analyzed mammary quarters was calculated by averaging all the pixels within the defined area, while the minimum and maximum temperatures were determined by automatically identifying the points with lowest temperature (cold spot) and highest temperature (hot spot), respectively.

### 2.4. Collection of Milk Samples and Measurement of Body Temperature

Milk sample collection occurred between 07:45 a.m. and 06:00 p.m. on a monthly basis over 14 months, totaling 1035 samples. Dirtiness scores were assigned to the mammary glands [18] before any procedures. Pre- and post-milking sanitizations were performed individually, with the automatic attachment and removal of teat cups. Sanitization was conducted using an iodine-based disinfectant, followed by backflushing and air drying. The electrical conductivity (EC) of the milk was measured using sensors in the milk line of each teat cup. A 60 mL sample was collected from each mammary quarter for somatic cell count (SCC) and fat content measurement by flow cytometry (CombiFoss 7, Foss, Hillerod, Denmark) and Fourier-transform infrared spectroscopy, respectively.

Prior to the collection of milk samples to investigate the infectious etiology of mastitis, automatic pre-milking teat sanitization was performed, the first three milk jets from each mammary quarter were discarded, and the front and rear right-side teats were sanitized with cotton soaked in 70% ethanol (v/v) until they were visually clean [19]. Clinical mastitis cases were investigated in all mammary quarters using a dark-bottomed strip cup immediately after discarding the first jets. Samples for microbiological diagnosis were collected in duplicate in sterilized test tubes and sent for laboratory analysis.

After milking, the cows were guided to a restraint alley to measure body temperature by inserting a digital clinical thermometer with an audible alarm indicating temperature stability into the rectum.

### 2.5. Laboratory Analyses and Definition of Mammary Gland Health Status

Microorganisms were identified based on the macroscopic observation of colonies, according to their morphotinctorial, biochemical, and culture characteristics [20]. The samples were plated on Petri dishes containing 5% defibrinated sheep blood agar, incubated under aerobic conditions at 37°C, and maintained for up to 72 h, with plate readings conducted at 24-hour intervals.

A mammary quarter was classified as “positive” for the presence of microorganisms when one or more identical colonies with the same morphology, pigmentation, and type of hemolysis were isolated. If three or more types of colonies were isolated, the sample was classified as contaminated [19]. Following isolation, the microorganisms were cryopreserved in BHI broth with glycerol and sent for analysis using matrix-assisted laser desorption ionization time-of-flight mass spectrometry (MALDI-TOF MS) [21]. *Escherichia coli* was used as a positive control in the analyses. After extraction procedures, the samples were analyzed using MicroFlex 3.4 equipment (Bruker Daltonik, Bremen, Germany), and mass spectrum processing was performed with the MALDI Biotyper software, Version 4.1.70 (Bruker Daltonik, Bremen, Germany), for microorganism identification (MBT Version 7311 MPS Library).

The obtained results were expressed in scores, with ≥ 1.7 considered reliable for genus identification and ≥ 2.0 reliable for both genus and species identification [21]. If a microorganism was not identified in the first MALDI-TOF MS reading, the protocol was repeated. Isolated microorganisms were grouped according to their capacity to induce an increase in SCC (Table 1).

Given the possibility of obtaining SCC results automatically through robotic milking, the health status of the mammary quarter was defined exclusively based on SCC, where values equal to or below 200,000 cells/mL were considered as healthy mammary glands, while values above this threshold were classified as SCM [3, 23, 24].

Microbiological isolation was used to develop an analytical attribute with the aim of assessing its impact on the predictive characteristics of SCM and determining the best way to incorporate it into the execution of the XGBoost algorithm. To this end, the isolation results were grouped into negative vs. positive and compared with the grouping based on Table 1 (negative vs. major pathogens, minor pathogens, and others). This analysis was conducted to facilitate decision making regarding the optimal strategy for algorithm optimization.

### 2.6. Statistical Analysis

#### 2.6.1. Data Preparation for Analysis

Initially, the data were prepared for analysis through a pre-selection process that involved removing missing data gaps to optimize and ensure the quality and accuracy of subsequent analyses. Incomplete or erroneous records were identified and removed from the dataset to be subjected to analysis. A total of 744 datasets related to SCM diagnostic observations (75%) were randomly reserved for the “training” phase to build prediction models, and 247 sets (25%) were allocated for the “testing” phase to evaluate model performance and ensure sample representativeness, totaling 991 datasets. In this phase, quantitative and qualitative value transformations and data normalization were also performed since the attribute value scales varied in some decimal places, necessitating numerical values to be assigned to categorical variable data. The “dummy_columns” and “scale” functions were utilized for the appropriate corrections and adaptations of observations.

#### 2.6.2. Hyperparameter Adjustment for Model Optimization

The XGBoost algorithm was selected for data analysis due to its high scalability, as it is based on a tree boosting framework that uses significantly fewer resources. The computational efficiency of XGBoost allows for the construction of highly accurate models within a reduced time frame and under limited computational capacity [11]. The algorithm exhibits great flexibility by enabling adjustments across a wide range of hyperparameters [12], which allows for the optimization of model accuracy for the classification problem addressed in this work. Compared to other algorithms, XGBoost offers notable advantages, such as internal regularization capability that minimizes the risk of overfitting, making it more robust in the presence of heterogeneous variables, and it has a proven track record of success in solving problems in the medical field, surpassing other decision tree–based algorithms [12].

The calculations were performed on a computer running Windows 11 Professional 64 bit, equipped with an Intel i7 processor with hyper-threading of 8 cores operating at 4.9 GHz and 32 GB of RAM. The XGBoost machine learning algorithm was selected for generating the predictive model. The R software, Version 4.2.1 (R Core Team, 2022, https://www.r-project.org/), was used in conjunction with the RStudio development interface, Version 2023.09.0 build 463, for algorithm development and execution.

The “xgboost” package, Version 1.7.5.1, was adopted, and through the “expand.grid” and “train()” functions, the hyperparameters Learning Rate (“eta”), Minimum Sum of Weights (“min_child_weight”), Maximum Depth of a Tree (“max_depth”), Control the Sample's Proportion (“subsample”), Column Sample by Tree (“colsample_bytree”), and Minimum Loss Reduction (“gamma”) were tuned as per Table 1 of the supporting information. The other hyperparameters were kept at their default values. A maximum number of 1000 trees has been set, and the number of unbiased variables is based on “ncol(*x*) − 1.” The *k*-fold cross-validation technique was used to evaluate the performance of the generated models and improve their generalization capability, with the number of resampling iterations (“numbers”) set to 10 and the number of repeats (“repeats”) set to 5.

**Figure 2:**
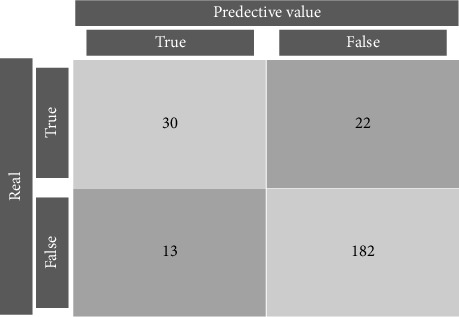
Confusion matrix obtained with the optimized XGBoost algorithm using all of the thermographic data.

In order to ensure result repeatability, the command “set.seed(123)” was defined for the algorithm. For each model, the data were randomly divided according to the dependent variable “mastitis” (binary variable “0” representing “healthy mammary glands” vs. “1” representing “mammary glands with SCM”) using the “createDataPartition” function. The “confusionMatrix” function was set to “everything” to generate maximum predictive characteristics for evaluating the model's performance, as depicted in Figure 2.

#### 2.6.3. Attributes Used for Predictive Model Optimization

The used attributes were grouped as follows to adjust the algorithm:

i. Environment-related attributes: AT, RH, and THI.
ii. Animal-related attributes: “Breed” (“Breed Hol” for pure Holstein animals vs. “Breed mixed” for Holstein vs. Jersey crossbred animals), “Body temperature,” teat cleanliness (“Dirtiness score”); “Parity”; lactation stage (“Days in milk”), which was divided into three phases: 0–90 days postpartum (early lactation), 91–245 days (mid), and after 246 days postpartum (late); and position of the mammary gland of the right antimer (“Mammary gland position”), where “Mammary gland_A” corresponded to the anterior mammary gland and “Mammary gland_P” to the posterior mammary gland;
iii. Milk-related attributes: milk yield of the mammary gland (“Milk yield”), “EC,” and milk fat content (“Milk fat”).
iv. Thermographic attributes: coldest point in the mammary gland ROI (“Cold”), hottest point in the ROI (“Hot”), average temperature of the ROI (“Average”), thermal amplitude of the ROI (“HotCold”), and the difference between the average temperature of the mammary gland ROI and the animal's body temperature (“AvgBT”).
v. “Microbiological evaluation.” This latter attribute was used in two different forms of grouping: 1st—positive microbiological isolation (“Pathogen_P”) vs. negative (“Pathogen_N”); and 2nd—negative isolation (“Pathogen_N”) vs. group of Major Pathogens (“Pathogens_Major”) vs. group of Minor Pathogens (“Pathogens_Minor”) vs. Other Pathogens (“Pathogens_Other”).

The most relevant variables for solving the classification problem were identified and selected for constructing the final model with the best fit and performance. The best predictors were then subjected to the test set.

#### 2.6.4. Evaluation of the Predictive Model

The model's performance was assessed through calculations of the following predictive characteristics of bovine SCM: accuracy (ACU), sensitivity (Se), specificity (Sp), precision, F1-Score, and AUC [4]. ACU corresponded to the probability of mammary quarters being correctly classified out of the total quarters, as described in the equation: ACU = (TP + TN)/(TP + TN + FP + FN). The Se, also known as “recall,” corresponded to the probability of true positives (TP) out of the total mammary quarters actually diagnosed with SCM, as described in the equation: Se = TP/(TP + FN). The Sp, on the other hand, corresponded to the probability of true negatives (TN) out of the total healthy mammary quarters, as described in the equation: Sp = TN/(TN + FP). Precision corresponded to the probability of TP out of the total mammary quarters classified with SCM and is described in the following equation: Precision = TP/(TP + FP), while the F1-Score combined precision and Se as per the equation: F1-Score = (2 × Precision × Se)/(Precision + Se).

The model's performance was primarily evaluated using AUC scores (95% confidence interval) in combination with Se and Sp values to understand overall performance and choose attributes that, when combined or excluded, showed higher values in these characteristics, ranging from 0 to 1.

Correlation analysis between variables was conducted through determination of the Pearson *r* coefficient, where values from 0 to 0.3 were considered negligible, 0.31 to 0.5 were considered weak, 0.51 to 0.7 moderate, 0.71 to 0.9 strong, and greater than 0.9 were considered very strong [25].

Cohen's kappa index was used to analyze the performance of the models by assessing the degree of agreement between the predicted and actual classifications derived from the confusion matrices generated by the attribute combinations. This index aimed to evaluate the model's accuracy, considering the chance of correct predictions occurring by random chance, and to determine which combinations provide more reliable predictions and improve performance. To achieve this, the programming function “kappa2()” was used, with results expressed on a scale from 1 (perfect agreement) to −1 (complete disagreement), where 0 represents agreement equivalent to chance [26].

## 3. Results and Discussion

During the study, the AT varied between 13.0°C and 33.1°C, with an average of 25.3°C, and the RH ranged from 24.3% to 90.6%, with an average of 64.0%. The THI varied between 58.0 and 80.5, with an average of 73.2. The SCM was detected in 21.7% of the analyzed udders.

The most common microorganisms were *S. aureus* and *S. chromogenes* (30.4% and 24.5%, respectively), highlighting the relevance of these agents in the epidemiology of the disease. In order of importance, the following were isolated: *S. dysgalactiae* (8.0%), *S. epidermidis* (4.5%), *S. saprophyticus* (4.0%), *S. uberis* (3.0%), Other CNS (3.0%), *S. warneri* (2.7%), *L. lactis* (2.7%), *S. hyicus* (1.8%), *Corynebacterium* spp. (1.8%), *S. simulans* (1.3%), and *E. cloacae* (1.3%). The remaining microorganisms had isolation rates below one percentage point.

Adjustments to prediction models by appropriately weighting the variables can improve predictive performance and maximize data usage, as some variables may not be informative [5, 23]. Thus, initially, attributes were selected that, when executed together, yielded the best performance for decision making with XGBoost. This selection was based on the values of predictive characteristics and importance graphs generated by the model. Subsequently, the importance of thermographic data for the algorithm's performance in diagnosing SCM was analyzed.

Given the numerous possibilities of combinations using the available attributes, the decision was made to study their individual contribution within the groups, starting with the environment-related variables. To this end, all variables were used during algorithm execution, and initially, within the environment-related attribute group, five different combinations were adopted (Table 2).

The AUC is a widely used metric for evaluating binary data classification problems and has the benefit of being independent of result rates [23]. However, in the present study, a balance between Se and Sp results was also sought. This aimed to ensure that prioritizing one predictive metric would not compromise the performance of another. It was observed that the use of the THI presented the highest AUC value among the combinations of environment-related attributes.

**Figure 3:**
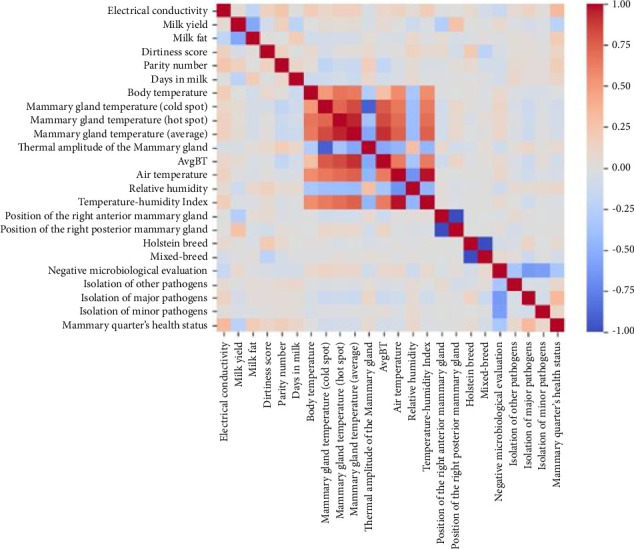
Heatmap of the Pearson correlation matrix for the attributes used in XGBoost optimization.

Environmental characteristics can interfere with the efficiency of IRT. The influence of the THI on IRT results has been documented previously, highlighting the relationship between high THI values and increased body temperature in dairy cows, as well as a positive correlation with surface temperatures measured by IRT, including the udder [27]. Similarly, in the present study, the highest correlations of udder surface temperatures recorded by IRT were associated with AT and THI (classified as moderate to strong; Table 3). All Pearson correlations between the attributes and the health status of the mammary gland can be observed in Figure 3.

**Figure 4:**
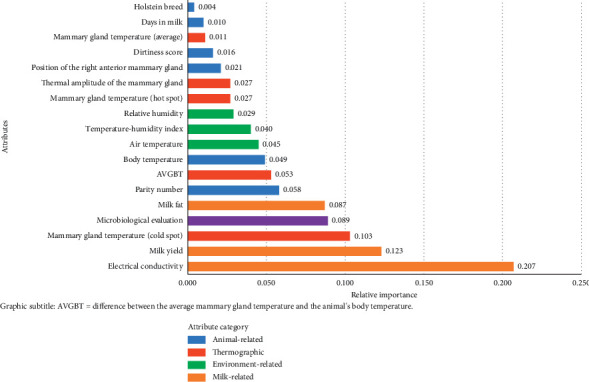
Relative importance of the attributes for decision making with XGBoost executed using all variables.

The environment-related attributes were relevant for decision making, although they did not comprise the main group of importance (Figure 4). Among these, both the THI and AT can be prioritized in optimizing the algorithm.

Due to the better AUC result observed with the use of the THI and its relative importance (Figure 4), it was decided to prioritize the use of this attribute over the others associated with the environment to advance in optimizing the algorithm. Subsequently, combinations with animal-related attributes were adopted (Table 4).

The relevance of the animal-related attributes in XGBoost execution for SCM diagnosis became evident when, upon excluding all of them in the analysis, the lowest values of predictive characteristics were obtained. The importance of parity and lactation stage has been previously reported using neural networks [23].

When variables were excluded in the analyses, there was no improvement in AUC values compared to maintaining all animal-related attributes. However, excluding the attributes related to “Parity,” “Body temperature,” and mammary gland position resulted in losses in this predictive characteristic. The same was not observed with the exclusion of the “Dirtiness score” attribute, where the highest AUC value (0.835) was obtained, providing the best Se result (57.69%) observed up to that point.

Body temperature showed a moderate correlation with the “Cold” (0.507), “Hot” (0.666), and “Average” (0.647) variables, while all other animal-related attributes showed low-magnitude correlations (below 0.2) with the thermographic variables. Therefore, only the “Dirtiness score” attribute was excluded to continue with the remaining analyses.

Next, the XGBoost algorithm was executed using different forms of classification of the attribute related to microbiological isolation, obtaining the results shown in Table 5.

It was noted that the incorporation of the microbiological evaluation into the set of attributes executed with the XGBoost algorithm was important to increase Se, although there was also an improvement in all other predictive characteristics. When classifying the microorganisms into major, minor, and other pathogens, the best AUC result was obtained (0.843), along with all other predictive characteristics. The relevance of major pathogens influencing techniques like IRT was also observed previously by Velascos-Bolaños [24]. The authors studied Holstein cows in tropical environments using IRT and found that the presence of major pathogens was able to increase the surface temperature of the udder by 1.16°C.

Subsequently, the analyses using the algorithm included the following set of attributes: environment-related (“THI”); animal-related (variable referring to mammary gland position, breed of the animal, “Parity,” “Days in milk,” and “Body temperature”); microbiological evaluation, separating into major pathogens, minor pathogens, and other pathogens; milk-related (“EC,” “Milk fat,” and “Milk yield”); and thermographic (“Cold,” “Hot,” “HotCold,” “Average,” and “AvgBT”). Afterward, the influence of the milk-related variables was studied by removing one of the following attributes at a time: “Milk fat,” “Milk yield,” and “EC,” which resulted in the outcomes presented in Table 6.

All milk-related attributes proved to be relevant for optimizing the XGBoost algorithm. Removing any of them resulted in a decrease in values for almost all measured predictive characteristics. Different milk characteristics have also been reported to influence the effectiveness of machine learning models for predicting SCM in dairy cows [3, 28].

For instance, EC is an important attribute as a predictive factor for SCM, as it increases with changes in milk ion composition due to elevated levels of Na^+^ and Cl^−^. Additionally, SCM significantly reduces milk volume, with an apparent increase in fat percentage and EC elevation [3]. In the present study, we observed that healthy mammary glands showed averages of 3.29 L of milk production per milking, 1.55% of fat, and 4.08 mS/cm of EC, while those with SCM produced 2.53 L of milk per milking, with 2.17% of fat, and 4.45 mS/cm of EC.

In this study, the EC consistently remained the main criterion of importance for the algorithm's decision making, regardless of the combination of attributes. Other relevant criteria were milk production and fat percentage, although their correlations with SCM incidence (0.325; −0.228, and 0.197, respectively) can be classified individually as weak. Similar to the results obtained herein, milk EC was the first classification criterion between udders with SCM and healthy ones in a thermographic study with Holstein cows in Turkey, using a decision tree algorithm [1].

Cows with SCM showed a negative and high-magnitude correlation (−0.96) between milk production and udder surface temperature measured by IRT, although the same was not observed in healthy udders (0.16) [29]. When we ran the XGBoost algorithm without the “Milk yield” variable, there was a reduction in values for all predictive characteristics, especially Se.

With the algorithm's attribute combination base already defined after previous analyses and its known predictive characteristics (0.843 of AUC; 57.7% of Se; 93.3% of Sp; 69.8% of precision; 63.2% of F1-Score; and 85.8% of ACU), we proceeded to assess the influence of the thermographic attributes in the diagnosis of SCM. To this end, the XGBoost algorithm was executed with the combinations shown in Table 7.

Comparing prediction model performances between studies that use very disparate datasets and different frameworks for diagnosing mastitis proves to be challenging. However, it is important to highlight the results achieved and reported in dairy cows by other researchers and in different locations [28].

It was observed that using only thermographic data for diagnosing SCM, excluding all other attributes, was not ideal. The lowest results were obtained under this condition for most of the predictive characteristics, notably with low AUC (0.527) and Se (13.5%). In another study using IRT alone and with SCC as a diagnostic screening technique, it was pointed out that IRT is a method that does not satisfactorily identify SCM, with Se results similar to those in the present study, ranging from 19.0% to 64.4% depending on the milking method and the cutoff point for the average udder surface temperature, while Sp ranged from 60.0% to 98.6% and the AUC between 58.8% and 62.2% [24]. However, there were increases in the values of the predictive characteristics when using all or some thermographic attributes.

**Figure 5:**
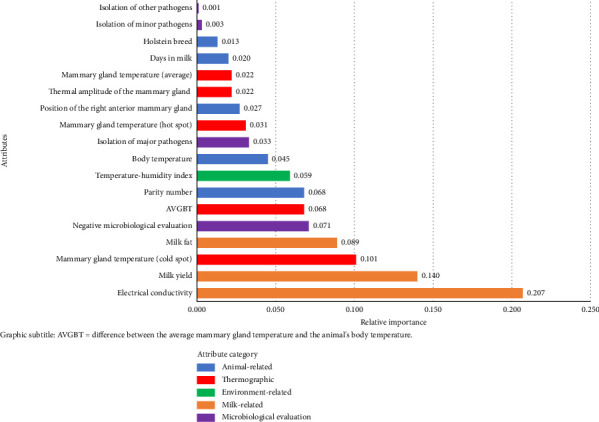
Relative importance of attributes when running XGBoost after optimizing the model with all thermographic variables.

In the importance plot generated from running the XGBoost algorithm with all thermographic variables (Figure 5), the relevance of the “Cold” attribute can be noted, which was considered the most important attribute obtained by IRT for decision making.

**Figure 6:**
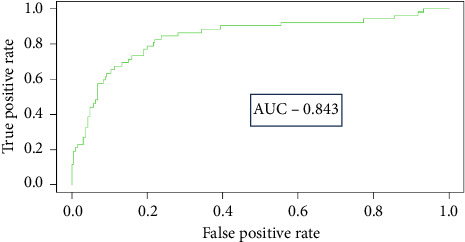
AUC obtained with the optimized XGBoost algorithm using all of the thermographic data for the diagnosis of SCM in lactating cows.

The selection of thermographic variables or the combination of variables to be used is relevant to optimize the performance results of the models. While our study identified the coldest point in the mammary gland ROI as the most important attribute, other authors have used the average udder temperature [24] or emphasized greater relevance for the hottest point [29]. This variation can occur according to environmental conditions (more intense cold, mild temperatures, or warmer temperatures), as the coldest point of the mammary gland, the hottest point, or the average temperature can alternate their position in the importance ranking. The use of the combination of all thermographic attributes provided a better AUC (0.843; Figure 6) and greater Sp (93.3%).

In a similar study conducted by Bobbo [23], the Se results ranged from 38.1% to 61.6%, classified by the authors as low to moderate. The Sp rates, on the other hand, were considered relatively high, exceeding 82%. In all attribute combinations with which XGBoost was executed in the present study, the Sp rates remained high and varied relatively little (from 89.74% to 93.5%), while the variations in Se ranged from 13.46% to 59.62%.

Zhou et al. [4] reported Se rates that did not exceed 85%, and the obtained Sp of 77.8% was considered poor by these authors. Conversely, Coskun and Aytekin [1] described higher Se (90.2%) than our study, similar AUC (0.853), and lower Sp (80.4%). These authors observed that 77.6% of cows with udder surface temperatures below 38.6°C were healthy, and 58.6% with temperatures above this limit had SCM.

The results obtained herein demonstrate that the use of XGBoost was able to improve the diagnostic capacity of SCM when IRT was employed. However, implementing IRT in the routine of the farm involves other factors. The choice of data to be used in the models should also consider the incorporation of environmental monitoring techniques, zootechnical control, and diagnostics in the production system [28]. Computerized herd management systems have been implemented by modern farms, allowing variables such as milk production and EC to be automatically recorded during milking and used in mastitis alarm systems [3]. On the other hand, data such as parity and the lactation stage of cows, which are recorded in farm management systems, are easy to use [28].

In a study with dairy cows monitored by automated systems, eight algorithms were used to compare the generated data, and it was observed that XGBoost showed low capacity in differentiating healthy cows from cows with health problems (metritis, clinical mastitis, hoof problems, and digestive disorders) due to a Se of 58.8%, although the model achieved the third-best performance (0.828 AUC). This same study reported the following values for other diagnostic characteristics: 80.6% Sp, 73.6% ACU, 58.8% precision, and the same value for the F1-Score [4]. In our work, aiming at the diagnosis of SCM, we achieved similar values for diagnostic characteristics in the optimized model. However, the variations in these metric results, particularly Se, depending on the attribute set used, highlight the importance of incorporating data from new technologies to achieve better outcomes.

**Figure 7:**
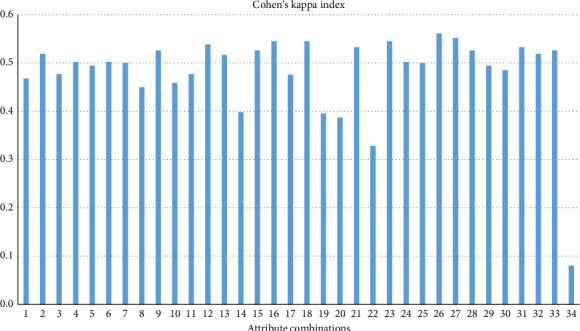
Cohen's kappa index values according to the attribute combinations used in the execution of the XGBoost algorithm.

In our work, the Cohen's kappa index results obtained from the different attribute combinations showed agreement ranging from 0.080 to 0.561, as observed in Figure 7. In nearly all attribute combinations in our study, the quality of the model predictions was similar to that observed by Bobbo et al. [23], who reported values ranging from 0.362 to 0.502 across various prediction models used for diagnosing SCM.

The lowest kappa value observed in our study (0.08) was obtained when only thermographic attributes were used in executing XGBoost, indicating a lower agreement between the predicted and actual classifications when using IRT alone as a diagnostic tool associated with machine learning under our experimental conditions. On the other hand, the second lowest kappa value (0.328) was observed when all milk-related attributes were excluded from the analysis, underscoring their importance for predicting SCM, as also evidenced by the analysis of other metrics.

The high Sp of XGBoost, using all thermographic data, makes it a promising confirmation mechanism for SCM. Its high ability to identify mammary glands free from the disease makes it a valuable tool for further evaluation of cows previously selected as positive by more sensitive screening methods. The fate of false-positive animals can be reduced through our optimized model, minimizing incorrect sanitary or zootechnical management decisions. XGBoost presents itself as a promising tool to reduce false alerts in automated milking systems when the goal is the early detection of SCM. Its application avoids wasting time and resources on healthy cows, allowing attention to be focused on animals that truly need it.

To develop the capability for automated diagnosis of SCM in robotic milking systems, it is essential to incorporate data from various sources that contribute to quality attributes. We highlighted the ability of IRT to produce relevant attributes for algorithmic decision making, which impacts its predictive capacity. Although currently IRT requires time, equipment costs, and a better understanding of how its results interact with other variables, such as environmental conditions (temperature and humidity) or the circadian cycle of the dairy cow, this technique has significant potential for automation.

Incorporating IRT into automatic milking systems can be facilitated through the development of thermographic sensors that lower the cost of the technique and accelerate data acquisition. However, the complexity of interactions between IRT data and other variable data will require ongoing advancements in machine learning for increasingly accurate diagnosis of bovine SCM.

For the use of IRT in other applications, such as clinical diagnosis of human diseases, there is concern regarding the standardization of conditions, especially environmental ones. We understand that fully exploiting the potential of the technique and its application in dairy cattle involves understanding IRT's behavior under as many natural conditions as possible. Consequently, this robust dataset highlights that IRT and machine learning algorithms, such as XGBoost, are complementary and inseparable tools.

## 4. Conclusions

The IRT proved to be a value-enhancing tool for diagnosing bovine SCM through machine learning. However, the XGBoost algorithm, when fed exclusively with data from the studied thermographic attributes, was not able to satisfactorily differentiate between diseased and healthy mammary glands. On the other hand, data analysis by XGBoost combined with other diagnostic tools yielded promising results, increasing the efficiency of mastitis diagnosis. The model adjusted using the combined results of IRT and the XGBoost algorithm showed potential for confirming the diagnosis of bovine SCM.

The development of sensors for real-time acquisition of thermographic images and the use of artificial intelligence for the automated interpretation of thermograms can help make their use in robotic milking feasible. However, the use of IRT for the diagnosis of bovine SCM should consider other factors, such as specialized labor, equipment costs, and available time for the technique to be performed.

## Figures and Tables

**Table 1:** Classification of isolated microorganisms (adapted from Kirkeby et al. [22]).

Major pathogens	Minor pathogens	Other pathogens
*Escherichia coli*	*Corynebacterium* spp.	*Streptococcus* spp.
*Staphylococcus aureus*	*Corynebacterium bovis*	*Staphylococcus hyicus*
*Streptococcus dysgalactiae*	*Staphylococcus auricularis*	*Streptococcus penaeicida*
*Streptococcus uberis*	*Staphylococcus capitis*	*Bacillus megaterium*
	*Staphylococcus chromogenes*	*Bacillus pumilus*
	*Staphylococcus epidermidis*	Catalase-negative cocci
	*Staphylococcus saprophyticus*	*Deinococcus fícus*
	*Staphylococcus simulans*	*Enterobacter cloacae*
	*Staphylococcus warneri*	*Enterococcus faecalis*
	*Staphylococcus xylosus*	Gram-negative bacilli
	Other CNS	*Lactococcus lactis*
		*Lysinibacillus fusiformis*
		*Micrococcus luteus*
		*Nocardia farcinica*
		*Rothia endophytica*
		Yeast

Abbreviations: CNS = Coagulase-negative *staphylococci* SCC = somatic cell count.

**Table 2:** XGBoost algorithm performance according to the use of environment-related attributes in six predictive characteristics of SCM in lactating cows.

Combinations	AUC	Se	Sp	Precision	F1-Score	Accuracy
1	0.8280	0.5000	0.9282	0.6500	0.5652	0.8421
2	0.8230	0.5577	0.9282	0.6744	0.6105	0.8421
3	0.8270	0.5000	0.9333	0.6667	0.5714	0.8543
4	0.8340	0.5385	0.9282	0.6667	0.5957	0.8502
5	0.8230	0.5192	0.9333	0.6750	0.5870	0.8340

*Note:* Combinations: 1—only air temperature; 2—only relative humidity; 3—association between air temperature and relative humidity; 4—only the THI; 5—without the use of environment-related data; AUC—area under the receiver operating characteristic curve.

**Table 3:** Pearson correlations between environmental and thermographic variables in diagnostic evaluations of SCM in lactating cows.

Attributes	AT	RH	THI	Cold	Hot	Average	HotCold	AvgBT
AT	1.000	−0.660	0.970	0.656	0.698	0.747	−0.464	0.634
RH	—	1.000	−0.463	−0.395	−0.400	−0.416	0.292	−0.345
THI	—	—	1.000	0.647	0.693	0.746	−0.455	0.638
Cold	—	—	—	1.000	0.721	0.827	−0.911	0.769
Hot	—	—	—	—	1.000	0.940	−0.371	0.826
Average	—	—	—	—	—	1.000	−0.549	0.911
HotCold	—	—	—	—	—	—	1.000	−0.539
AvgBT	—	—	—	—	—	—	—	1.000

*Note:* Attributes: Cold = coldest point of the mammary gland; Hot = hottest point of the mammary gland; Average = average temperature of the mammary gland; HotCold = thermal amplitude of the mammary gland; AvgBT = difference between the average temperature of the mammary gland and body temperature. The results of the correlations between all variables were statistically significant (*p* < 0.001).

Abbreviations: AT = air temperature, RH = relative humidity, and THI = temperature-humidity index.

**Table 4:** XGBoost algorithm performance according to different combinations of animal-related attributes in six predictive characteristics of SCM in lactating cows.

Combinations	AUC	Se	Sp	Precision	F1-Score	Accuracy
6	0.8340	0.5385	0.9282	0.6667	0.5957	0.8462
7	0.8320	0.5577	0.9179	0.6444	0.5979	0.8421
8	0.8080	0.5000	0.9179	0.6190	0.5532	0.8300
9	0.8350	0.5769	0.9231	0.6667	0.6186	0.8502
10	0.8220	0.5000	0.9231	0.6341	0.5591	0.8340
11	0.8340	0.5000	0.9333	0.6667	0.5714	0.8421
12	0.8200	0.5577	0.9385	0.7073	0.6237	0.8583
13	0.8290	0.5769	0.9179	0.6522	0.6122	0.8462
14	0.8040	0.4808	0.8974	0.5556	0.5155	0.8097

*Note:* Combinations: 6—using all animal-related attributes; 7—removing only the “Breed” attribute; 8—removing only the “Mammary gland position” attribute; 9—removing only the “Dirtiness score” attribute; 10—removing only the “Parity” attribute; 11—removing only the “Days in milk” attribute; 12—removing only the “Body temperature” attribute; 13—using the attributes related to “Mammary gland position,” “Parity,” and “Body temperature”; 14—removing all animal-related attributes; AUC—area under the receiver operating characteristic curve.

**Table 5:** XGBoost algorithm performance according to the microbiological culture–based classification in six predictive characteristics of SCM in lactating cows.

Combinations	AUC	Se	Sp	Precision	F1-Score	Accuracy
15	0.8350	0.5769	0.9231	0.6667	0.6186	0.8502
16	0.8430	0.5769	0.9333	0.6977	0.6316	0.8583
17	0.7720	0.5192	0.9231	0.6429	0.5745	0.8381

*Note:* Combinations: 15—using microbiological culture, grouping the results into positive isolation vs. negative isolation; 16—using microbiological culture, grouping into: negative isolation, major pathogens group, minor pathogens group, and other pathogens group; 17—excluding microbiological culture from the set of attributes to be executed; AUC—area under the receiver operating characteristic curve.

**Table 6:** XGBoost algorithm performance according to different combinations of milk-related attributes in six predictive characteristics of SCM in lactating cows.

Combinations	AUC	Se	Sp	Precision	F1-Score	Accuracy
18	0.8430	0.5769	0.9333	0.6977	0.6316	0.8583
19	0.8090	0.4038	0.9385	0.6364	0.4941	0.8259
20	0.8220	0.4231	0.9231	0.5946	0.4944	0.8178
21	0.8400	0.5962	0.9179	0.6596	0.6263	0.8502
22	0.7260	0.3461	0.9333	0.5806	0.4337	0.8097

*Note:* Combinations: 18—using all milk-related attributes; 19—removing the “EC” attribute; 20—removing the “Milk yield” attribute; 21—removing the “Milk fat” attribute; 22—removing all milk-related attributes; AUC—area under the receiver operating characteristic curve.

**Table 7:** XGBoost algorithm performance according to different combinations of thermographic attributes in six predictive characteristics of SCM in lactating cows.

Combinations	AUC	Se	Sp	Precision	F1-Score	Accuracy
23	0.8430	0.5769	0.9333	0.6977	0.6316	0.8583
24	0.8260	0.5385	0.9282	0.6667	0.5957	0.8462
25	0.8220	0.5577	0.9179	0.6444	0.5979	0.8421
26	0.8330	0.5962	0.9333	0.7045	0.6458	0.8623
27	0.8400	0.5962	0.9282	0.6889	0.6392	0.8583
28	0.8400	0.5769	0.9231	0.6667	0.6186	0.8502
29	0.8270	0.5769	0.9231	0.6667	0.6186	0.8502
30	0.8370	0.5192	0.9282	0.6585	0.5806	0.8421
31	0.8350	0.5962	0.9179	0.6596	0.6263	0.8502
32	0.8280	0.5577	0.9282	0.6744	0.6105	0.8502
33	0.8190	0.5192	0.9333	0.6750	0.5870	0.8462
34	0.5270	0.1346	0.9282	0.3333	0.1918	0.7611

*Note:* Combinations: 23—using all thermographic attributes; 24—removing all thermographic attributes; 25—using only the thermographic attribute “Cold”; 26—using only “Cold” and “AvgBT”; 27—using only “Cold,” “AvgBT,” and “HotCold”; 28—using “Cold,” “AvgBT,” “HotCold,” and “Hot”; 29—using only “Average”; 30—using only “HotCold”; 31—using only “AvgBT” and “HotCold”; 32—using only “Hot”; 33—using only “AvgBT”; 34—using only the thermographic attributes and excluding others; AUC—area under the receiver operating characteristic curve.

## Data Availability

The data that support the findings of this study are available from the corresponding author upon reasonable request.

## Supporting Information

Table 1 of the supporting information describes the values of the hyperparameters used to obtain the best results based on the attribute combination employed in the execution of the XGBoost algorithm.

## References

[1] Coskun G., Aytekin I. (2021). Early Detection of Mastitis by Using Infrared Thermography in Holstein-Friesian Dairy Cows via Classification and Regression Tree (CART) Analysis. *Selcuk Journal of Agricultural and Food Sciences*.

[2] Sharma P., Bhardwaj K., Wadhwa D. R., Katoch S. (2020). Subclinical Mastitis and Its Effect on Milk Components in Crossbred Cows. *Himachal Journal of Agricultural Research*.

[3] Ebrahimie E., Ebrahimi F., Ebrahimi M., Tomlinson S., Petrovski K. R. (2018). A Large-Scale Study of Indicators of Sub-Clinical Mastitis in Dairy Cattle by Attribute Weighting Analysis of Milk Composition Features: Highlighting the Predictive Power of Lactose and Electrical Conductivity. *Journal of Dairy Research*.

[4] Zhou X., Xu C., Wang H. (2022). The Early Prediction of Common Disorders in Dairy Cows Monitored by Automatic Systems With Machine Learning Algorithms. *Animals*.

[5] Xudong Z., Xi K., Ningning F., Gang L. (2020). Automatic Recognition of Dairy Cow Mastitis From Thermal Images by a Deep Learning Detector. *Computers and Electronics in Agriculture*.

[6] Olasehinde O. (2021). Infrared Thermography and Machine Learning in Livestock Production. *International Journal of Advanced Research*.

[7] Satheesan L., Kittur P. M., Alhussien M. N., Lal G. S., Kamboj A., Dang A. K. (2024). Reliability of Udder Infrared Thermography as a Non-Invasive Technology for Early Detection of Sub-clinical Mastitis in Sahiwal (*Bos indicus*) Cows under Semi-Intensive Production System. *Journal of Thermal Biology*.

[8] Sathiyabarathi M., Jeyakumar S., Manimaran A. (2016). Infrared Thermography: A Potential Noninvasive Tool to Monitor Udder Health Status in Dairy Cows. *Veterinary World*.

[9] Sinha R., Bhakat M., Mohanty T. K. (2018). Infrared Thermography as Non-Invasive Technique for Early Detection of Mastitis in Dairy Animals-A Review. *Asian Journal of Dairy and Food Research*.

[10] Lokhorst C., De Mol R. M., Kamphuis C. (2019). Invited Review: Big Data in Precision Dairy Farming. *Animal*.

[11] Chen T., Guestrin C. Xgboost: A Scalable Tree Boosting System.

[12] Budholiya K., Shrivastava S. K., Sharma V. (2022). An Optimized XGBoost Based Diagnostic System for Effective Prediction of Heart Disease. *Journal of King Saud University-Computer and Information Sciences*.

[13] da Costa A. N. L., Feitosa J. V., Montezuma P. A., de Souza P. T., de Araújo A. A. (2015). Rectal Temperatures, Respiratory Rates, Production, and Reproduction Performances of Crossbred Girolando Cows Under Heat Stress in Northeastern Brazil. *International Journal of Biometeorology*.

[14] Pereira A. R., Angelocci L. R., Sentelhas P. C. (2002). Agrometeorologia: Fundamentos e Aplicações Práticas. Guaíba: Agropecuária. https://www.scribd.com/document/517527747/Pereira-Et-Al-2002-Agrometeorologia-Fundamentos-e-Aplicacoes-Praticas-OCRrc.

[15] Thom E. C. (1959). The Discomfort Index. *Weatherwise*.

[16] Romanello N., de Brito Lourenço Junior J., Barioni Junior W. (2018). Thermoregulatory Responses and Reproductive Traits in Composite Beef Bulls Raised in a Tropical Climate. *International Journal of Biometeorology*.

[17] Hovinen M., Siivonen J., Taponen S. (2008). Detection of Clinical Mastitis With the Help of a Thermal Camera. *Journal of Dairy Science*.

[18] Cardozo L. L., Thaler Neto A., Souza G. N. (2015). Risk Factors for the Occurrence of New and Chronic Cases of Subclinical Mastitis in Dairy Herds in Southern Brazil. *Journal of Dairy Science*.

[19] Oliver S. P., Lewis M. J., Gillespie B. E., Dowlen H. H., Jaenicke E. C., Roberts R. K. (2004). *Microbiological Procedures for the Diagnosis of Bovine Udder Infection and Determination of Milk Quality*.

[20] Koneman E. W., Allen S. D., Janda W. M., Schreckenberger P. C., Winn Junior W. C. W. (2001). *Diagnóstico Microbiológico-Texto e Atlas Colorido*.

[21] Barcelos M. M., Martins L., Grenfell R. C. (2019). Comparison of Standard and On-Plate Extraction Protocols for Identification of Mastitis-Causing Bacteria by MALDI-TOF MS. *Brazilian Journal of Microbiology*.

[22] Kirkeby C., Toft N., Schwarz D. (2020). Differential Somatic Cell Count as an Additional Indicator for Intramammary Infections in Dairy Cows. *Journal of Dairy Science*.

[23] Bobbo T., Biffani S., Taccioli C., Penasa M., Cassandro M. (2021). Comparison of Machine Learning Methods to Predict Udder Health Status Based on Somatic Cell Counts in Dairy Cows. *Scientific Reports*.

[24] Velasco-Bolaños J., Ceballes-Serrano C. C., Velásquez-Mejía D. (2021). Application of Udder Surface Temperature by Infrared Thermography for Diagnosis of Subclinical Mastitis in Holstein Cows Located in Tropical Highlands. *Journal of Dairy Science*.

[25] Mukaka M. M. (2012). Statistics Corner: A Guide to Appropriate Use of Correlation Coefficient in Medical Research. *Malawi Medical Journal*.

[26] Sreeja N. K. (2018). A Weighted Pattern Matching Approach for Classification of Imbalanced Data With a Fireworks-Based Algorithm for Feature Selection. *Connection Science*.

[27] Peng D., Chen S., Li G., Chen J., Wang J., Gu X. (2019). Infrared Thermography Measured Body Surface Temperature and its Relationship With Rectal Temperature in Dairy Cows under Different Temperature-Humidity Indexes. *International Journal of Biometeorology*.

[28] Pakrashi A., Ryan C., Guéret C. (2023). Early Detection of Subclinical Mastitis in Lactating Dairy Cows Using Cow-Level Features. *Journal of Dairy Science*.

[29] Khakimov A. R., Pavkin D. Y., Yurochka S. S., Astashev M. E., Dovlatov I. M. (2022). Development of an Algorithm for Rapid Herd Evaluation and Predicting Milk Yield of Mastitis Cows Based on Infrared Thermography. *Applied Sciences*.

