# Supplementary material for: Machine Learning Techniques Associated With Infrared Thermography to Optimize the Diagnosis of Bovine Subclinical Mastitis
Source: Vet Med Int. 2025 Feb 8;2025:5585458. doi: 10.1155/vmi/5585458 (PMC11830110; doi:10.1155/vmi/5585458)
Supplement: Supporting Information — Additional supporting information can be found online in the Supporting Information section. [file 5585458.f1.docx]

**Supplementary material**

**Table 1.** Hyperparameters tuned for analyses with different combinations of attributes used for optimizing XGBoost algorithm results.

| Combinations | eta | min_child_weight | max_depth | subsample | colsample_bytree | gamma |
| --- | --- | --- | --- | --- | --- | --- |
| 1 | 0.1 | 3 | 8 | 0.5 | 1 | 3 |
| 2 | 0.1 | 1 | 8 | 0.75 | 1 | 3 |
| 3 | 0.1 | 1 | 8 | 1 | 1 | 3 |
| 4 | 0.01 | 3 | 8 | 0.75 | 1 | 1 |
| 5 | 0.1 | 3 | 8 | 0.75 | 1 | 3 |
| 6 | 0.01 | 3 | 8 | 0.75 | 1 | 1 |
| 7 | 0.01 | 3 | 8 | 0.75 | 1 | 1 |
| 8 | 0.1 | 3 | 8 | 0.75 | 1 | 3 |
| 9 | 0.001 | 1 | 8 | 0.75 | 1 | 1 |
| 10 | 0.1 | 1 | 6 | 0.75 | 1 | 1 |
| 11 | 0.01 | 3 | 8 | 0.75 | 1 | 3 |
| 12 | 0.1 | 1 | 8 | 0.75 | 1 | 3 |
| 13 | 0.1 | 3 | 2 | 1 | 1 | 3 |
| 14 | 0.1 | 3 | 2 | 1 | 1 | 3 |
| 15 | 0.001 | 1 | 8 | 0.75 | 1 | 1 |
| 16 | 0.1 | 1 | 8 | 0.75 | 1 | 3 |
| 17 | 0.1 | 1 | 8 | 0.75 | 1 | 3 |
| 18 | 0.1 | 1 | 8 | 0.75 | 1 | 3 |
| 19 | 0.1 | 1 | 6 | 1 | 1 | 3 |
| 20 | 0.1 | 3 | 8 | 0.75 | 1 | 1 |
| 21 | 0.1 | 3 | 8 | 0.75 | 1 | 1 |
| 22 | 0.1 | 3 | 8 | 1 | 1 | 3 |
| 23 | 0.1 | 1 | 8 | 0.75 | 1 | 3 |
| 24 | 0.1 | 1 | 6 | 1 | 1 | 3 |
| 25 | 0.1 | 3 | 8 | 1 | 1 | 3 |
| 26 | 0.1 | 3 | 8 | 1 | 1 | 3 |
| 27 | 0.1 | 1 | 6 | 1 | 1 | 3 |
| 28 | 0.1 | 3 | 8 | 1 | 1 | 1 |
| 29 | 0.1 | 1 | 8 | 1 | 1 | 3 |
| 30 | 0.1 | 1 | 8 | 0.75 | 1 | 1 |
| 31 | 0.1 | 1 | 6 | 1 | 1 | 3 |
| 32 | 0.1 | 1 | 6 | 1 | 1 | 3 |
| 33 | 0.1 | 1 | 6 | 1 | 1 | 3 |
| 34 | 0.001 | 3 | 2 | 0.75 | 1 | 1 |

Hyperparameters: Learning Rate (“eta”), Minimum Sum of Weights (“min_child_weight”), Maximum Depth of a Tree (“max_depth”), Control the Sample’s Proportion (“subsample”), Column Sample by Tree (“colsample_bytree”) and Minimum Loss Reduction (“gamma”).
